# The Factor Structure of the Mood Disorder Questionnaire in Tunisian Patients

**DOI:** 10.2174/1745017902016010082

**Published:** 2020-07-30

**Authors:** Uta Ouali, Lamia Jouini, Yosra Zgueb, Rabaa Jomli, Adel Omrani, Fethi Nacef, Antonio Preti, Mauro Giovanni Carta

**Affiliations:** 1 Department of Psychiatry, Razi Hospital, Manouba, Tunisia; 2 Faculty of Medicine of Tunis, University of Tunis El Manar, Tunis, Tunisia.; 3 Centre de Compétences en Psychiatrie et Psychothérapie, Pôle de Psychiatrie et Psychothérapie, Hôpital du Valais (HVS)- Centre Hospitalier du Valais Romand, Switzerland.; 4 Tunisian Bipolar Forum, Erable Médical Cabinet 324, Tunis, Tunisia.; 5 Center for Liaison Psychiatry and Psychosomatics, University Hospital, University of Cagliari, Cagliari, Italy.

**Keywords:** Bipolar disorder, Hypomania/mania, Mood Disorder Questionnaire, Confirmatory factor analysis, Early detection, Factorial validity

## Abstract

**Background:**

The Mood Disorder Questionnaire (MDQ) is a frequently used screening tool for the early detection of Bipolar Disorder (BD), which is often unrecognized or misdiagnosed at its onset. In this study, data from Tunisia has been used to evaluate the psychometric properties of the Arabic MDQ.

**Methods:**

The sample included 151 patients with a current major depressive episode. The Arabic adapted version of the Structured Clinical Interview for DSM-IV-TR was used to formulate a diagnosis, yielding 62 patients with BD and 89 with unipolar Major Depressive Disorder (MDD). Principal component analysis with parallel analysis was used to establish the spontaneous distribution of the 13 core items of the MDQ. Confirmatory Factor Analysis (CFA) was used to check the available factor models. Receiver Operating Characteristic (ROC) analysis was used to assess the capacity of the MDQ to distinguish patients with BD from those with MDD.

**Results:**

Cronbach’s α in the sample was 0.80 (95%CI: 0.75 to 0.85). Ordinal α was 0.88. Parallel analysis suggested two main components, which explained 59% of variance in the data. CFA found a good fit for the existing unidimensional, the two-factor, and the three-factor models. ROC analysis showed that at a threshold of 7, the MDQ was able to distinguish patients with BD from those with MDD with extraordinary negative predictive value (0.92) and a positive diagnostic likelihood ratio of 3.8.

**Conclusion:**

The Arabic version of the MDQ showed good measurement properties in terms of reliability, factorial validity and discriminative properties.

## 1 INTRODUCTION

Bipolar disorder is a severe condition with estimated lifetime prevalence ranging from 1 to 5% across countries [1], and high risk of functional impairment and suicide [2]. Bipolar disorder is often unrecognized or misdiagnosed at its onset, since it may begin with a depressive episode that can be indistinguishable from unipolar depression and because hypomania or sub-threshold hypomania can go undetected or be attributed to substance use [3, 4]. Several screening measures have been developed to favour the early identification of bipolar spectrum disorders [5]. The Mood Disorder Questionnaire (MDQ) [6], is one of the most frequently used tools for the screening of bipolar spectrum disorders [7]. The core of the MDQ includes 13 yes/no questions on symptoms related to mania/hypomania (Table **1**), plus two additional questions on the simultaneous occurrence of the symptoms and the impact of the symptoms on work, family life, legal troubles or getting into fights.

The psychometrics properties of the MDQ have been extensively investigated [8, 9]. At the conventional agreed cut-off of 7, the MDQ has a good capacity to detect people with probable bipolar disorder, with sensitivity around 0.60 and specificity around 0.85 [9]. Diagnostic accuracy of the MDQ did not differ significantly between studies from Eastern and Western countries, when taking into account clinical confounding variables [8, 9]. Overall, good accuracy of the MDQ was found in clinical psychiatric settings in the United Kingdom [10], in Italy [11], in France [12], in Spain [13, 14], and in Turkey [15], as well as in community samples in Italy [16] and France [17]. Accuracy was of a lower level in the United States in both the clinical setting [18], and in prison [19].

The MDQ items cover a broad range of content, from increased energy and risky behavior to distractibility and racing thoughts. The specificity of mania/hypomania of the symptomatic range covered by the MDQ has been questioned, since some of these symptoms may also occur in anxiety, in trauma-related, and in impulse control disorders [20]. However, some of this criticism may be misguided, since they overlook the high comorbidity of bipolar spectrum disorders with anxiety [21, 22], trauma-related [23], and impulsivity/dyscontrol disorders [24, 25]. Nonetheless, whether the MDQ taps into a single factor of mania/hypomania or whether it can be decomposed into different dimensions is still a matter of debate.

Several studies based on Exploratory Factor Analysis (EFA) provided evidence for two factors of the MDQ. However, the studies did not agree on the distribution of items by factor and on its meaning [26]. By taking into account several independent EFA Chinese studies, Massida *et al.* [26] identified three dimensions of the MDQ, labeled as “acceleration” (including items 1, 2, 4, 5, 6, and 7), “energy” (items 3, 8, 9, and 10), and “imprudence” (items 11, 12, and 13). This partition is the most coherent with a similar three-domain structure of mania, composed by a dimension of mental activation (including racing thoughts), a dimension of elated/high mood (including elevated/expansive mood), and a dimension of behavioral activation (including overactivity) [27]. However, the most commonly described structure of the MDQ purports a two-factor structure. Mangelli *et al*. [28], in an Italian sample of 1034 individuals recruited from a community *via* advertisement, have found two factors with EFA, one is the elated mood and increased energy (items 1, 3, 4, 8, 9, 10, and 11) and another is the racing thoughts, distractibility and risky behavior (items 2, 5, 6, 7, 12, and 13). Carta *et al*. [29], in a study including 291 participants, provided evidence with Confirmatory Factor Analysis (CFA) for two factors, a “self-confidence and energy” factor (items 3, 8, 9, 10, and 11) and a “acceleration, danger and irritability” factor (items 1, 2, 5, 6, 7, 10, 12, and 13). Item 10 “much more social” loaded on both factors, and item 4 “less sleep” was excluded from the CFA on the basis of a past EFA, which included 2278 Italian participants sampled from several Italian towns. Stanton and Watson [7] applied exploratory structural equation modeling (ESEM) to the MDQ responses of 700 participants reporting current psychiatric treatment and enrolled via the Amazon Mechanical Turk (presumably from the United States, but this is unclear). They found evidence for two factors, one is the “positive activation” (items 3, 4, 5, 8, and 9), and the other is “negative activation” (sic) (items 1, 2, 5, 6, 7, 10, 12, 13). Item 5 “more talkative than usual” was allowed to cross-load on both factors, while item 11 “more interested in sex than usual” did not load on any of the two factors. The factor structure of the MDQ is still confusing and the models that have been reported in the literature are still not reproduced so far as independent samples. Overall, there is some evidence that the MDQ might in fact have a one-dimensional structure (see CFA of the unidimensional model and Rasch analysis in Carta *et al*. [29]).

Thus far, most studies on the factor structure of the MDQ have been carried out in European or Anglo-Saxon cultures. This is the first study to investigate the diagnostic validity and the factorial structure of the Arabic version of the MDQ. We also tested the discriminant validity of the Arabic version of the MDQ concerning its ability to delimitate patients with BD from patients with MDD, which is nuclear for its use as a first-step screening tool in patients with mood disorders.

## 2 METHODS

The study has been conducted according to the guidelines of the 1995 Declaration of Helsinki and its revisions [30]. The Institutional Review Board (IRB) of Razi Hospital has approved the study protocol (authorization signed on 8 Oct 2014).

### 2.1 Participants

Patients were recruited between February 2015 and August 2018 at the Department of Psychiatry A of Razi Hospital La Manouba, Tunisia. All consecutive patients who arrived at the enrollment center complaining about depression were invited to take part in the study. Patients were included in the study when the clinician formulated a diagnosis of a current major depressive episode. Thereafter, all included patients were enrolled for the Tunisian Arabic adapted version of the Structured Clinical Interview for DSM-IV-TR (SCID) to confirm the diagnosis of Major Depressive Episode and to ascribe the episode to a unipolar or bipolar mood disorder. In particular, patients were diagnosed as BD when the Tunisian Arabic SCID interview revealed one or more past episode(s) of hypomania or mania according to DSM*-*IV*-*TR criteria. When no past episode of hypomania or mania according to DSM*-*IV*-*TR criteria could be identified in the anamnesis of the patient, the resulting diagnosis was MDD single episode or recurrent unipolar MDD. Additional inclusion criteria were: aged between 18 and 65 years old; and the capacity of providing informed consent. Exclusion criteria were: illiteracy or other cause of inability to read; documented history of mental retardation; and cognitive decline.

All included patients provided written informed consent.

### 2.2 Measures

The MDQ was translated into Standard Arabic language for this study and this Arabic version was used for the present investigation. Standard procedures were followed for the translation of the MDQ [31]. The MDQ was translated into Arabic language by a bilingual native editor, then back-translated into English by another bilingual native editor. The translation and back-translation were harmonized by the help of one independent researcher with a comprehensive knowledge of the tool. Cognitive debriefing with a pilot testing with six patients of the target population was arranged to identify potential issues or unclear terms. The results of the cognitive debriefing were used to finalize the translation with the help of all participants to the preceding phases of the translation.

### 2.3 Statistics

All data were coded and analyzed using the Statistical Package for Social Sciences (SPSS) version 20. Additional analyses were carried out in R [32].

All tests were two-tailed, with alpha set at p<0.05.

The means with standard deviations were reported for continuous variables. Counts and percentages were reported for categorical variables. Parametric or non-parametric tests, as appropriate, were used to compare continuous variables between groups. In the comparison of MDQ items by diagnosis, the effect sizes of the differences between diagnoses were calculated according to Cliff’s delta, which is appropriate in case of violations of normality. The Cliff’s delta represents the degree of overlap between the two distributions of scores and it ranges from –1 to +1 (according to the order of overlap between two groups). The threshold for judging the effect size with Cliff’s delta was: <0.15, negligible; between 0.16 and 0.48, moderate; ≥0.49, large.

Scale scores’ reliability was measured by *Cronbach’s alpha* or its ordinal version, which has a better fit for dichotomic items or for items showing skewness [33]. For group comparisons, reliability values of 0.70 are considered satisfactory [34]. However, if individual and important decisions must be made on the basis of reliability estimates, as is the case with screening or classificatory tools, values should be at least 0.90 [35].

#### 2.3.1 Exploratory and Confirmatory Factor Analysis

The data was preliminarily subjected to a Principal Component Analysis (PCA) to establish the spontaneous distribution of the 13 core items of the MDQ into one or more separate dimensions. Parallel analysis was used to determine the optimal number of components. In a parallel analysis, the scree plot of the observed data was compared with that of a random matrix of the same size as the original. The best solution is based on the number of components with eigenvalues higher than those generated by the random data, either simulated or resampled by permutation from the original data. The parallel analysis and the subsequent PCA were carried out with the psych package running in R [36]. Both were applied to a matrix of tetrachoric correlations, since we assumed, as in Carta *et al*. [29], that the binary responses result from the discretization of an intrinsically continuous latent structure (the manic/hypomanic syndrome).

Thereafter, CFA was applied to the data, by implementing some of the two-factor and three-factor models that were described in the literature, and precisely the three-factor model reported in Massida *et al*. [26], the two-factor model described by Mangelli *et al*. [28], and the alternative two-factor model reported by Carta *et al*. [29]. All models were compared to the simplest unidimensional model, which assumes all 13 core items of the MDQ tap into a single dimension of propensity to the manic/hypomanic syndrome.

Mardia’s test [37] revealed violation of multivariate normality in the data: small sample skew = 637, p < 0.0001. Therefore, the Diagonally Weighted Least Squares (DWLS) estimator was used. The DWLS approach automatically uses the WLS estimator with polychoric correlations as input to create the asymptotic covariance matrix. Parameters for fit estimation were: the chi-square, the Comparative Fit Index (CFI), the Root Mean Square Error of Approximation (RMSEA), and the Standardized Root Mean Square Residual (SRMR). RMSEA values of 0.08 or lower, SRMR values of 0.09 or lower, and CFI values of 0.90 or higher are considered acceptable [38]. McDonald's omega, as estimated from the model, was also reported [39]. McDonald's omega is a reliability coefficient with the advantage of taking into account the strength of association between items and constructs as well as item-specific measurement errors. Therefore, it provides more realistic estimates of the true reliability of the scale. McDonald's omega around 0.90 is considered acceptable. Model identification was verified following the Bekker *et al*. method [40]. According to this method, when the rank of the Jacobian matrix is the same as the number of free parameters in the factorial model, the model is identified. The calculation was based on a script available in lavaan version 0.5-16 or higher *et al*. [41].

#### 2.3.2 Receiver Operating Characteristic (ROC) Analysis

Receiver Operating Characteristic (ROC) analysis was used to assess the capacity of the MDQ to distinguish patients with BD from patients with MDD. The following information was reported to summarize the results of the ROC analysis: the area under receiver operator characteristic curve (AUC; with 95% confidence interval); sensitivity (the probability of a true positive case, **i.e.** probability of identifying a patient with BD); specificity (the probability of a true negative case, *i.e.* probability of identifying a patients without BD); positive predictive value (PPV, the probability that a person is a case of BD when a positive test result is observed) and negative predictive value (NPV, the probability that a person is not a case of BD when a negative test result is observed); positive diagnostic likelihood ratio (the odds ratio that a positive test result will be observed in a population of people with BD compared to the odds that the same result will be observed among a population of people without BD). AUC is considered good when ranging 0.80 to 0.90; it is considered fair or barely acceptable when ranging 0.70 to 0.80; below 0.70 is unacceptable. Sensitivity and specificity were used to derive the cut-off that best differentiated the patients with BD from those with MDD. To avoid the costs of false negatives (unrecognized BD) and false positives (un-necessary treatment of people without BD), values of sensitivity and specificity above 0.80 are optimal, between 0.70 and 0.80 are acceptable, and below 0.70 are poor and potentially harmful (see Maxim *et al*. [42]). Positive and negative predictive values are better understood in cohort studies and depend on the prevalence of the condition under test. As for the positive diagnostic likelihood ratio, the higher its value, the more useful is the test.

ROC analysis was conducted with the *pROC* package running in R [43].

The optimal cut-off point for the MDQ scores was established according to the Youden method [44], using the *Optimal Cutpoints* package running in R [45].

## 3 RESULTS

The sample included 151 patients with a current major depressive episode, 60 men (40%) and 91 women (60%). Mean age in the sample was 42 years (standard deviation [SD] = 9; range: 19 to 61 years old), with no difference by sex. The sample included 89 patients diagnosed with unipolar major depressive disorder (MDD; 59%), 62 diagnosed with a bipolar spectrum disorder, of whom 22 diagnosed with bipolar disorder type I (14.5%), 37 diagnosed with bipolar disorder type II (24.5%), and 3 diagnosed with Substance-induced Mood Disorder, type: manic (2%).

Overall, 69 patients had a university degree or higher education level (45.4%); the remaining patients (n=83; 54.6%) had a high school diploma or a lower level of education. There were 112 patients (74%) that reported being married; the remaining patients were single (n=27; 18%), divorced (n=10; 6%), widowed (n=1; 1%), or cohabiting with a partner (n=1; 1%).

Age of onset of the disorder was 33 years (SD=11; range: 12 to 58 years old), with no relevant differences by diagnosis.

Patients with bipolar spectrum disorders were more likely than patients diagnosed with MDD to have been admitted to a psychiatric hospital (n=22 [35.5% *versus* n=16 [18%]; χ^2^=5.05, p=0.025), to have attempted suicide (n=23 [37.7% *versus* n=15 [16.9%]; χ^2^=7.25, p=0.007), and to have a family history of bipolar disorder (n=16 [25.8% *versus* n=4 [4.6%]; χ^2^=12.24, p<0.0001). A family history of suicide attempt was also more likely in patients with bipolar spectrum disorders than in patients diagnosed with MDD, but the difference did not reach the pre-specified threshold for statistical significance (n=12 [19.4% *versus* n=8 [9.2%]; χ^2^=2.40, p=0.121).

### 3.1 Descriptive Statistics

Reliability, measured as internal consistency, was good: Cronbach’s aλπηα in the sample was 0.80 (95%CI: 0.75 to 0.85). Ordinal aλπηα was 0.88.

Positive replies on the core 13 items of the MDQ in the sample ranged from 33% (item 11, “much more interested in sex than usual”) to 79% (item 7, “easily distracted”). As expected for a mixed sample, there was a large dispersion by item, with a standard deviation ranging from 0.40 to 0.50 (Table **1**).

Total score on the MDQ ranged from 0 (n=1) to 13 (n=11), with mean = 7.2 (SD=3.3).

As expected, patients with bipolar spectrum disorders scored higher than patients diagnosed with MDD on the MDQ: 10.0±2.5 *vs* 5.2±2.3; t = 11.54; p <0.0001.

**Fig. (1):**
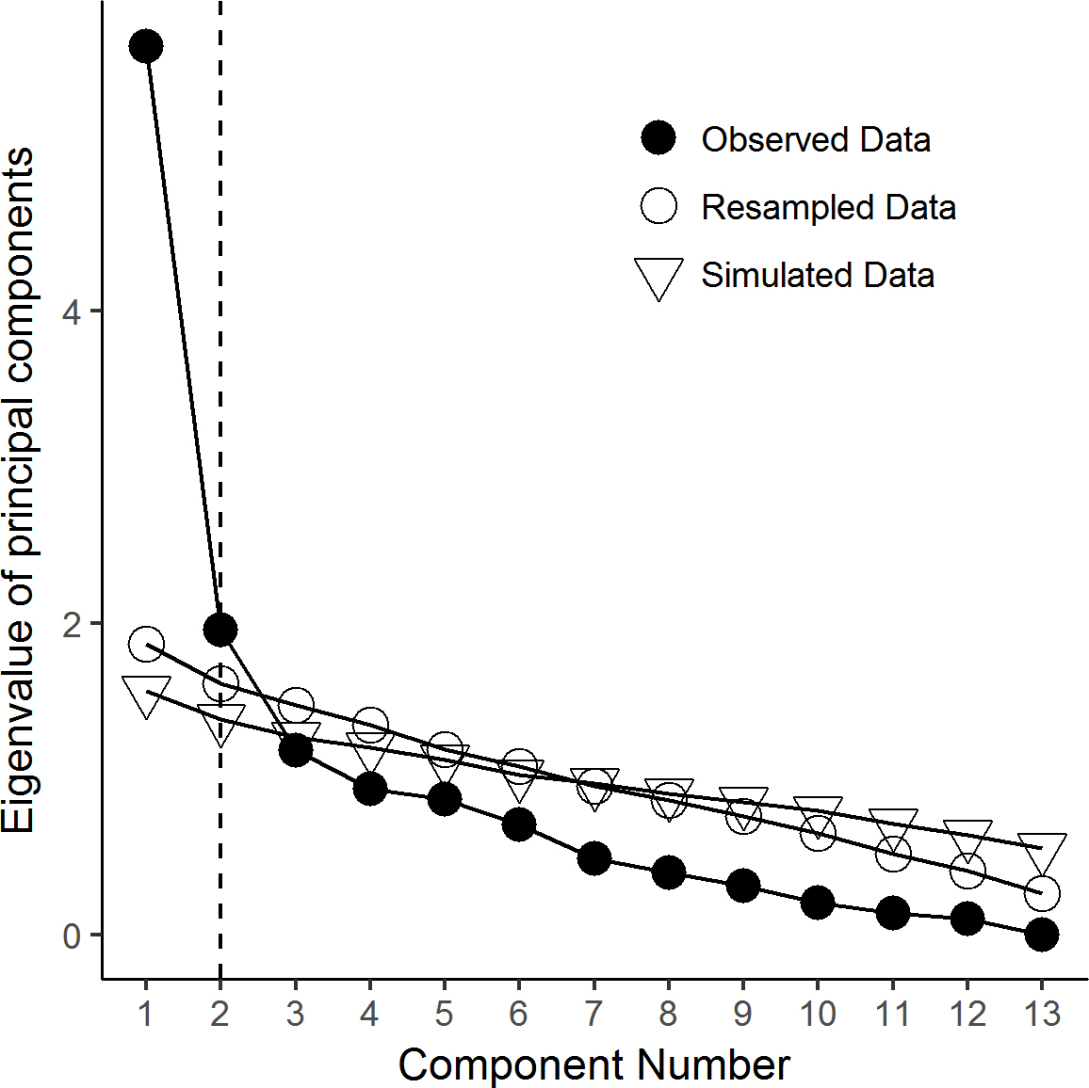
Profile plot of the distribution of the MDQ items’ scores by diagnosis. On the vertical axis (y-axis) it is reported the percentage of ‘yes’ endorsement of the item by diagnosis; on the horizontal axis (x-axis) there are the 13 items of the MDQ. Items for which a statistically significant difference at p<0.0001 was found on the Mann-Whitney U test were marked with an asterisk.

Overall, the response profile on the MDQ items of the patients diagnosed with MDD was clearly different from that of the patients diagnosed with BD (Fig. **1**).

The largest effect size for the differences between MDD and BD patients were found in items 1, 3, 8, 9, and 10. Overall, patients with MDD had the lowest chance of endorsement for the items 10 (“Much more social”), 11 (“Much more interested in sex”), and 12 (“Greater involvement in excessive, foolish or risky things”). Conversely, patients with BD had the highest chance of endorsement for items 8 (“Much more energy”), and 9 (“Much more active”).

In particular, patients with MDD scored lower than patients with BD on most MDQ items, with the exception of items 2 (“Being irritable”), 6 (“Thoughts raced “), and 7 (“Easily distracted”) (Table **2**).

### 3.2 Principal Component Analysis

**Fig. (2):**
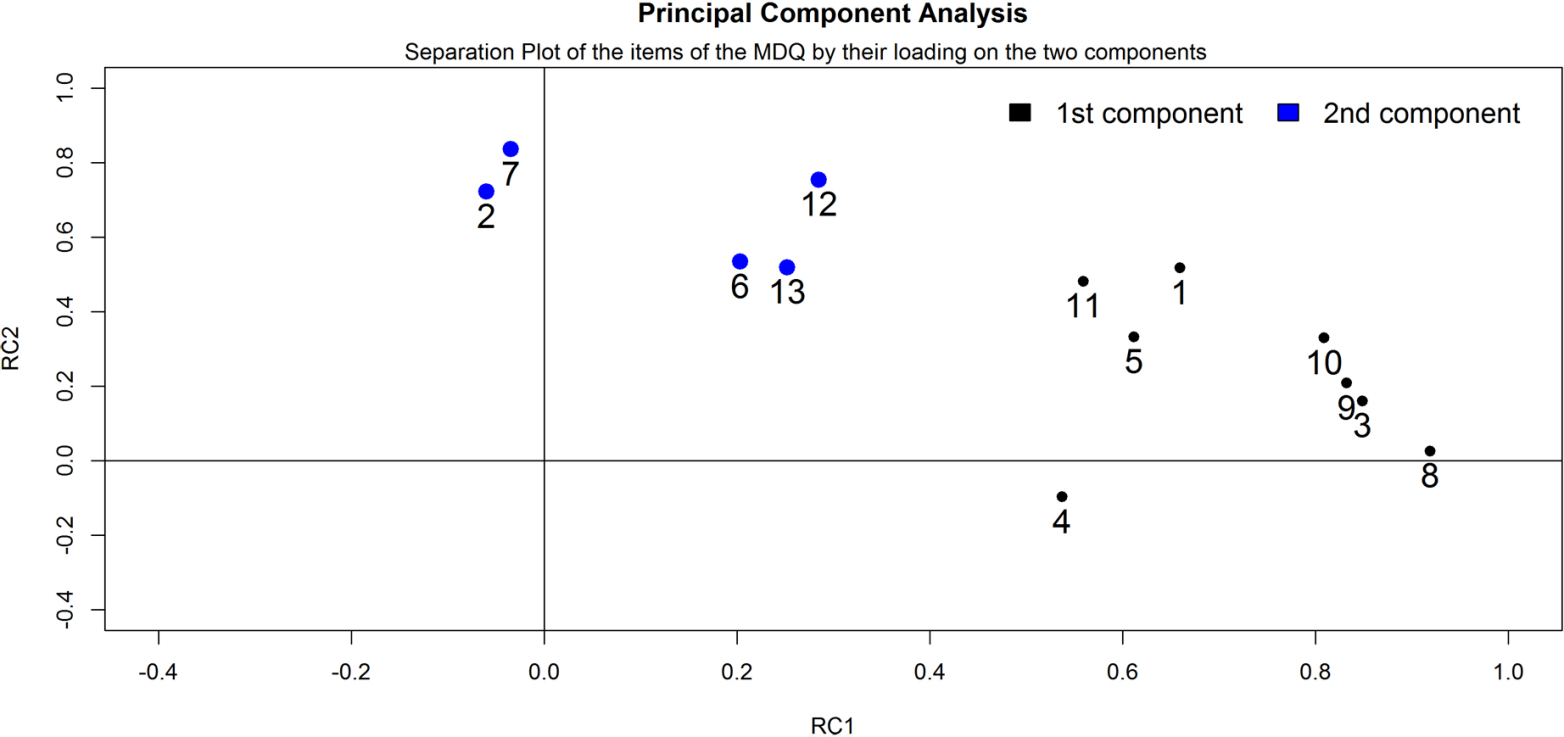
Parallel analysis applied to the items of the Mood Disorder Questionnaire (n = 13). Plot of the eigenvalues calculated on the basis of the actual data and of the simulated and resampled data. The number of dimensions to retain corresponds to the number of eigenvalues that have a higher value than the corresponding eigenvalues calculated on the basis of the simulated and resampled data. In this case, the number of dimensions to retain is 2 and is marked by a vertical dashed line.

Bartlett’s test of sphericity was 481.07 (p < 0.0001), Kaiser-Meyer-Olkin adequacy value was 0.80. The matrix, thus, can be factorized. In a parallel analysis, two components had eigen values superior to both simulated and resampled data (Fig. **2**).

The subsequent PCA with varimax rotation to maximize the separation of the components revealed that the two components explained 59% of the variance in the data (Table **3**).

The first component can be considered similar but not coinciding with the “elated mood and increased energy” dimension of Mangelli *et al*. [28], while the second component can be equated to the “racing thoughts, distractibility and risky behavior” dimension of Mangelli *et al*. [28].

**Fig. (3):**
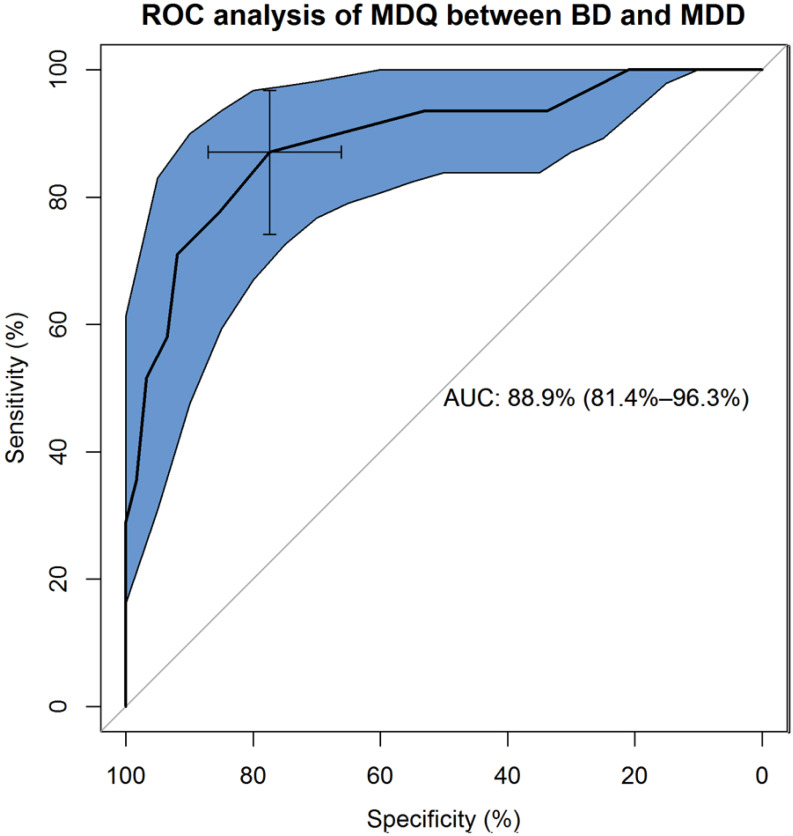
Separation plot of the results of the principal component analysis applied to the items of the Mood Disorder Questionnaire (n = 13). Items are plotted on the basis of their loadings on the two extracted main dimensions. An item is as much “separated” along the two dimensions as much its loading differs across the two dimensions, *i.e.*, it is very high on one dimension and very low on the other dimension.

Item 11 “Much more interested in sex” was less clearly separated between the two components (Table **3** and Fig. **3**).

### 3.3 Confirmatory Factor Analysis

Table **4** summarizes the fit of the models tested by CFA. All models were identified, and all models reached the threshold for a good fit for the CFI, the RMSEA, and the SRMR. McDonald’s omega was suboptimal (<0.90) for all models. In the CFA of the Carta *et al*. [29] two-factor model, item 10 had a poor loading on the factor it belongs to (*p* > 0.001), probably because of its cross-loading on both factors. For all other models, the items loaded on their factors with *p* < 0.0001.

Estimated factor loading was above 0.30 for 9 items out of 13 in the unidimensional model; for 10 items in the Massidda *et al*. [26] model; for 11 items in the Carta *et al*. [29] model (which allowed cross-loading); and for 11 items in the Mangelli *et al*. [28] model.

Overall, the models were similar to each other as far as indexes of fit were concerned, with the Massidda *et al*. (2016) [25] three-factor model showing marginally the best fit.

Conservatively, the MDQ in this sample can be considered unidimensional, thus its global score can be used for screening purposes.

### 3.4 Roc Analysis

**Fig. (4):**
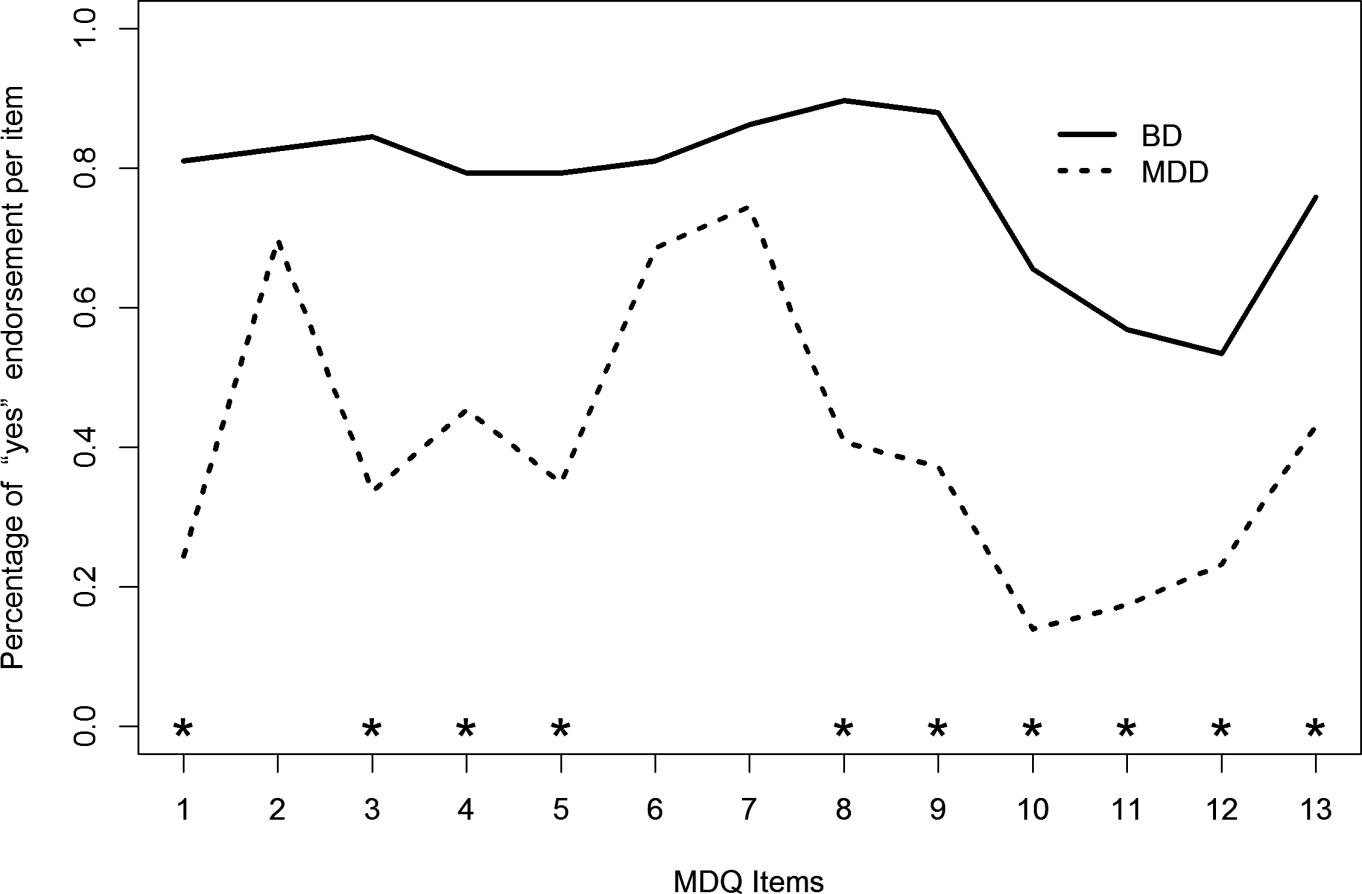
Receiver operator characteristic (ROC) curve of the predictive capacity of the MDQ in differentiating patients with BD from patients with MDD. Sensitivity and specificity are reported as percentages, with a cross indicating on the curve the best compromise between them (corresponding to the cut-off). The area under the ROC curve (AUC) is reported alongside its 95% confidence interval.

The MDQ had a good capacity of distinguishing patients diagnosed with BD from those diagnosed with MDD (Fig. **4**).

**Fig. (5):**
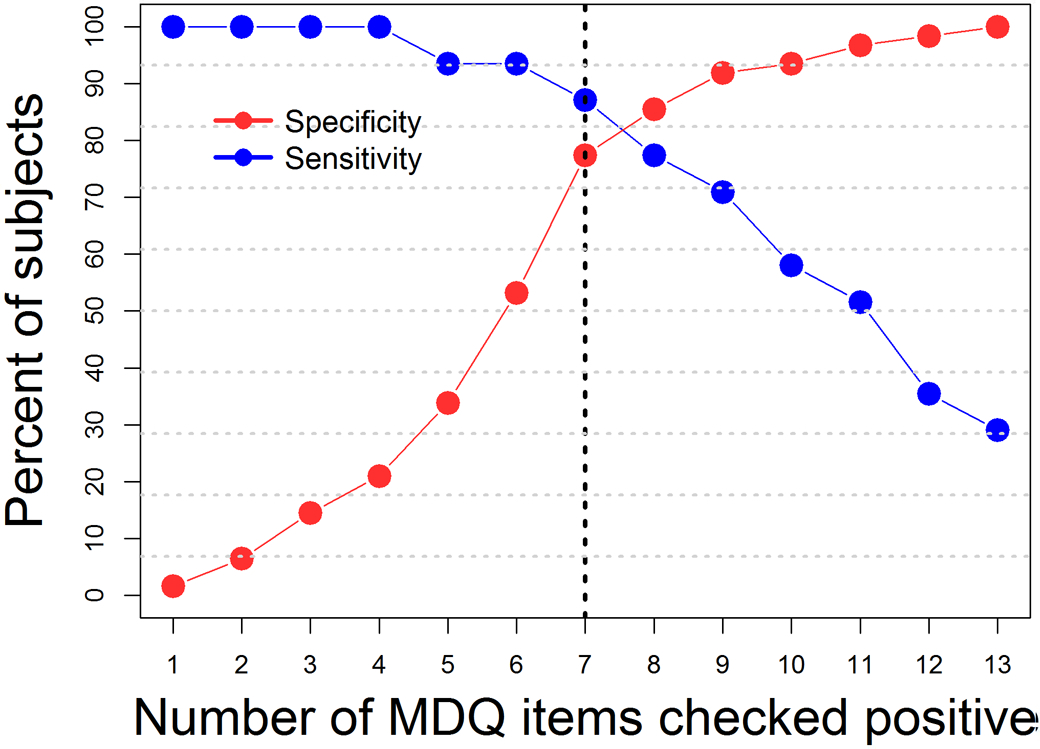
Operating characteristics of the MDQ for various threshold scores among 151 Tunisian patients diagnosed with a current episode of major depressive disorder either in the course of a unipolar or bipolar mood disorder as diagnosed with the SCID.

The AUC was good: 0.88 (95%CI: 0.81 to 0.96). Sensitivity was also good (0.87), while specificity was acceptable but not optimal (0.77). According to the Youden method, the suggested cut-off was 7, which represents a good compromise between sensitivity and specificity (Fig. **5**).

The positive predictive value was 0.66 and the negative predictive value was 0.92. The positive diagnostic likelihood ratio was 3.8, meaning that patients with a mood disorder scoring 7 or higher on the MDQ were about 4 times more likely to have a bipolar disorder than a major depressive disorder.

In the sample, 77 patients reached or surpassed the 7-point threshold on the MDQ, qualifying as a probable case of BD: 55 (out of 62; 89%) among those diagnosed with BD; 22 (out of 89; 25%) among those with MDD (odds ratio = 23.9 [95%CI: 9.5 - 60.2]; z = 6.75, df=1, p<0.0001).

## 4 DISCUSSION

This is the first study exploring the diagnostic validity and the factorial structure of the MDQ in an Arabic speaking sample of patients with mood disorders. The study provided evidence of good reliability of the Arab version of the MDQ, albeit with suboptimal values for a screening tool (<0.90; see Kottner *et al*. [35]). Reliability of the MDQ was surprisingly overlooked in past investigations. However, when tested, the Cronbach’s alpha appeared to be acceptable for group comparisons, with values ranging from 0.78 in a general population Hong Kong community sample [46], to 0.82 in Chinese patients diagnosed with mood disorders [47], 0.87 in a mixed sample of Brazilian patients with mood disorders and controls with no psychiatric disorder [48], 0.88 in Korean patients diagnosed with bipolar disorder [49], 0.89 in a French sample of patients diagnosed with MDD or bipolar spectrum disorders [12]. In this study, too, when tested with a procedure that is more suited for dichotomous (yes/no) data, reliability was 0.88. Nonetheless, when estimated from CFA with McDonald’s omega, reliability was still below the optimal threshold suggested for the screening tools [35]. Further investigation of the reliability of the MDQ is warranted. To the best of our knowledge, the only other screening tool for bipolar disorder so far validated in the Arabic language is the Arabic adaptation of the Hypomania Check List-32, second revision (HCL-32-R2) by Fornaro *et al*. [50], which shows a PPV for BD (I and II) of 0.93 and a NPV of 0.73. However, in the Fornaro study, the proportion of people with BD was inflated by active enrollment, therefore the information on the PPV and NPV should be taken with caution. Our study design attempted to represent “real life conditions”, as newly presenting patients were enrolled, while it was initially unknown whether their major depressive episode was uni- or bipolar.

In this study, the sensitivity of the MDQ was better (0.87) than in the past investigations: it was 0.69 in the meta-analysis of Carvalho *et al*. [8], and 0.76 in the meta-analysis of Wang *et al*. [9]. Specificity was 0.77 in this study, it was 0.79 in the meta-analysis of Carvalho *et al*. [8], and it was 0.88 in the meta-analysis of Wang *et al*. [9]. However, in primary care or general population settings, the sensitivity of the MDQ was calculated to be low (0.43), albeit with excellent specificity (0.95) [8]. This may depend on the tendency of people with hypomania to overlook their own symptoms and underreport them. Nevertheless, when compared with another frequently used screening tool for hypomanic/manic symptoms, the HCL-32, the MDQ revealed a better profile. In nine studies with available data, the two screening tools showed comparable sensitivity (0.82; 95%CI: 0.59-0.71 for the HCL-32, and 0.80; 0.71-0.86 for the MDQ), but the MDQ showed a better specificity (0.70; 0.59-0.71) than the HCL-32 (0.57; 0.48-0.66) [51]).

It should be noted that in this study, the large majority of the patients who were SCID diagnosed with a BD were detected as probable cases of BD by the MDQ at the suggested threshold of 7. However, about 25% of the patients SCID diagnosed with unipolar MDD screened positive for probable BD. As a matter of fact, when a patient presents with a major depressive episode, it cannot be excluded that it will manifest symptoms of hypomania or mania in the future. Indeed, a fraction of patients with unipolar MDD may switch to bipolar disorder over time, more often as an effect of antidepressant-associated mood-switching [52]. Moreover, subsyndromal and major depression are the known precursors of BD [53]. Thus, it cannot be excluded that a subsample of the patients SCID diagnosed with MDD in this study might have a propensity towards progressing into BD that was detected by the MDQ. Long-term longitudinal studies are necessary to test whether those patients with MDD who are detected as at risk of BD by the MDQ will evolve into a frank BD syndrome at follow-up.

As in past investigations (for a review, see Massidda *et al*. [26]), the data spontaneously distributed into two separated dimensions, one is elated mood and increased energy, and the other one is behavioral activation, secondary to racing thoughts and greater involvement into risky behavior. However, when CFA was applied to the data, a reasonable fit was found for several models of the MDQ. A marginally better fit was found for a three-factor model derived from studies that were done in China [26]. Indeed, a three-factor model is the most compatible with a similar three-dimension model of mania as composed by mental activation, elated/high mood, and behavioral activation [27]. Nevertheless, the unidimensional model also had a reasonably good fit to the data, as expected for a screening tool.

This is the first study to provide evidence for reproducibility of some of the models of MDQ factor structure that have been described in the literature. It should be noted that the sample was a mixed sample of patients diagnosed with affective disorders, including both patients diagnosed with MDD and patients with bipolar spectrum disorders. This may have led to some dispersion of the data, since patients with MDD, as expected, were much less likely to endorse the items of the MDQ. Moreover, the sample size was acceptable for principal factor analysis, with about 10 cases per item, but it was overall rather small for extended investigations of additional models (*e.g.*, the second-order, hierarchical and bifactor implementation of the models).

Some findings deserve attention. As in some non-Western studies [46-48, 54, 55], item 11 on a greater interest in sex was connected both to elated mood and to risky behavior. In all likelihood, there are patients that feel the increased sexuality that is related to hypomania/mania as troublemaking rather than egosyntonic, *i.e.*, acceptable to the needs and goals of the subject. Indeed, it cannot be excluded that the spontaneous distribution of the items into two dimensions at the PCA might be a reflection of the propensity of the patients of giving prominence to the more egosyntonic symptoms of hypomania/mania (those that grouped under the first dimensions of elated/high mood), or, conversely, of their tendency to principally report the most disturbing ones (those grouped under the second dimensions of racing thoughts and risky behavior). This may be related to the personal attitudes of the patients and their socio-cultural background rather than a real bipartition of the MDQ’s items. As a matter of fact, we agree with the consideration of Carta *et al*. [29], who considered that with such a small number of items, parsimony is preferable, thus favoring the unidimensional structure of the MDQ. Such a structure is coherent with the main purpose of the MDQ, which is not to measure the latent dimension of hypomania/mania along its continuum, but to identify which persons are located on its positive side, *i.e.*, to identify clinically at-risk subjects and discriminate them from those who do not have bipolar disorder.

## 5 LIMITATIONS

Small sample size for more complex analysis and the mixed nature of the sample represent the main limitations of the study. Convergent and divergent validity was not explored in this study, as it is customary for adaptations of questionnaires in another language (*e.g.*, see Ioannou *et al*. [56],). However, a ROC analysis was applied to assess the capacity of the MDQ to distinguish patients with BD from patients with MDD, and this is a more robust method to assess the discriminative validity of a test than simply a correlational analysis. Additional studies, with larger samples and from more diverse socio-cultural and ethnic populations are necessary for a full understanding of the factorial structure of the MDQ, in particular to explore the measurement invariance of the MDQ across diagnosis (BD *versus* MDD).

## CONCLUSION

The Arabic version of the MDQ showed good measurement properties in terms of reliability and factorial validity, suggesting that its items measure a single latent trait that clusters into two dimensions of elated mood and behavioral activation. In this study, the sensitivity and specificity of the MDQ were reasonably good, with a robustly informative diagnostic likelihood ratio of nearly 4.

The study is a contribution to a more comprehensive understanding of hypomania/mania across different ethnic and cultural enclaves, from the perspective of a real worldwide investigation of psychiatric disorders.

## Figures and Tables

**Table 1:** Descriptive statistics for the Mood Disorder Questionnaire items for the sample (n = 151).

Item	Mean *	SD	Skewness	Kurtosis
1. So hyper to get into trouble	0.47	0.50	0.11	-2.01
2. Irritable	0.75	0.43	-1.16	-0.64
3. More self-confident	0.54	0.50	-0.16	-1.99
4. Less sleep	0.59	0.49	-0.37	-1.88
5. More talkative	0.53	0.50	-0.11	-2.01
6. Thoughts raced	0.74	0.44	-1.08	-0.84
7. Easily distracted	0.79	0.40	-1.45	0.10
8. Much more energy	0.60	0.49	-0.43	-1.84
9. Much more active	0.58	0.49	-0.31	-1.92
10. Much more social	0.35	0.47	0.64	-1.60
11. Much more interested in sex	0.33	0.47	0.71	-1.51
12. Excessive, foolish or risky things	0.35	0.48	0.61	-1.64
13. Spending money got into trouble	0.56	0.49	-0.25	-1.96

^*^SD = Standard Deviation

^*^ For dichotomous items, item mean multiplied by 100 gives the percentage of “yes” responding.

**Table 2:** Distribution of scores for the Mood Disorder Questionnaire items by diagnosis.

	MDD n° = 89		BD n° = 62		Mann-Whitney U test	Cliff’s delta
Item	Mean *	SD	Mean *	SD		
1. So hyper to get into trouble	0.25	0.43	0.81	0.39	Z=-6.74, p<0.0001	**0.56**
2. Irritable	0.70	0.46	0.84	0.37	Z=-1.94, p=0.052	0.12
3. More self-confident	0.33	0.47	0.85	0.35	Z=-6.28, p<0.0001	**0.50**
4. Less sleep	0.47	0.50	0.81	0.39	Z=-4.19, p<0.0001	0.34
5. More talkative	0.34	0.47	0.80	0.40	Z=-5.59, p<0.0001	0.44
6. Thoughts raced	0.69	0.46	0.81	0.39	Z=-1.65, p=0.098	0.12
7. Easily distracted	0.75	0.43	0.84	0.37	Z=-1.22, p=0.223	0.11
8. Much more energy	0.40	0.49	0.90	0.29	Z=-6.16, p<0.0001	**0.49**
9. Much more active	0.37	0.48	0.87	0.33	Z=-6.10, p<0.0001	**0.51**
10. Much more social	0.13	0.34	0.66	0.47	Z=-6.64, p<0.0001	**0.51**
11. Much more interested in sex	0.17	0.37	0.55	0.50	Z=-4.89, p<0.0001	0.39
12. Excessive, foolish or risky things	0.23	0.42	0.55	0.50	Z=-4.02, p<0.0001	0.30
13. Spending money got into trouble	0.42	0.49	0.75	0.43	Z=-4.08, p<0.0001	0.33

MDD = Major Depressive Disorder; BD = Bipolar Disorder.

SD = Standard Seviation.

^*^ For dichotomous items, item mean multiplied by 100 gives the percentage of “yes” responding.

^**^ Large effect sizes were marked in bold.

**Table 3:** Principal component analysis of the Mood Disorder Questionnaire in the sample (n= 151).

Item	First component*	Second component*
1. So hyper to get into trouble	**0.66**	0.52
2. Irritable	-0.06	**0.72**
3. More self-confident	**0.85**	0.16
4. Less sleep	**0.54**	-0.10
5. More talkative	**0.61**	0.33
6. Thoughts raced	0.20	**0.53**
7. Easily distracted	-0.03	**0.84**
8. Much more energy	**0.92**	0.03
9. Much more active	**0.83**	0.21
10. Much more social	**0.81**	0.33
11. Much more interested in sex	**0.56**	0.48
12. Excessive, foolish or risky things	0.28	**0.75**
13. Spending money got into trouble	0.25	**0.52**
Proportion of variance	35%	24%

^*^Item is assigned to the component on which it has the highest loading

**Table 4:** Goodness-of-fit indices of the tested models.

	Goodness of fit indicators							Wald Rank Rule		
Model	*|^2^*	*df*	*p*	CFI	RMSEA (90%CI)	SRMR	McDonald’s ω	*n* columns	Rank	
Unidimensional model	95.96	65	0.008	0.96	0.058 (0.031 – 0.081)	0.082	0.81	26	26	Identified
Mangelli *et al*. (2005) two factor model	87.14	64	0.029	0.97	0.050 (0.017 – 0.075)	0.078	0.82	27	27	Identified
Carta *et al*. (2014) two factor model	77.52	52	0.012	0.97	0.059 (0.028 – 0.085)	0.080	0.82	26	26	Identified
Massidda *et al*. (2016) three factor model	81.44	62	0.050	0.98	0.047 (0.002 – 0.073)	0.076	0.82	29	29	Identified
Threshold for fit			*p* > 0.05	≥ 0.90	≤ 0.08	≤ 0.09	≥ 0.90	*n =*	Rank	
